# Oxidative Stress and Alveolar–Capillary Barrier Failure in Sepsis- and Severe Acute Pancreatitis-Associated ARDS

**DOI:** 10.3390/antiox15101368

**Published:** 2026-10-21

**Authors:** Sophia Rusch, Patricia Mester, Fabian Möller, Martina Müller, Karsten Gülow

**Affiliations:** Department of Internal Medicine I, Gastroenterology, Hepatology, Endocrinology, Rheumatology, Immunology, and Infectious Diseases, University Hospital Regensburg, 93053 Regensburg, Germany; sophia.rusch@ukr.de (S.R.); patricia.mester-pavel@klinik.uni-regensburg.de (P.M.); fabian.moeller@ukr.de (F.M.); martina.mueller-schilling@ukr.de (M.M.)

**Keywords:** acute respiratory distress syndrome (ARDS), oxidative stress, reactive oxygen species (ROS), sepsis, severe acute pancreatitis, mitochondrial dysfunction

## Abstract

Acute respiratory distress syndrome (ARDS) is the final common phenotype of diverse insults, but the route to alveolar–capillary barrier failure matters. Sepsis and severe acute pancreatitis provide an informative contrast: sepsis begins with infection and pathogen-associated molecular patterns, whereas pancreatitis is initially driven by sterile acinar-cell injury, necrosis and damage-associated molecular patterns, before infected necrosis can superimpose sepsis biology. These differences are clinically relevant since they determine whether redox-related signals must be interpreted in the context of pathogen burden and antimicrobial host defense or of initially sterile necrosis with subsequent risk of infected necrosis. In this review, we discuss how these distinct pathophysiological contexts engage NADPH oxidase (NOX)-derived oxidants, mitochondrial reactive oxygen species, reactive nitrogen species (RNS), lipid peroxidation, regulated cell death and impaired antioxidant defenses. We focus on the pulmonary endothelium and epithelium as compartments in which inflammatory and redox signals converge to disrupt barrier integrity, impair alveolar fluid clearance and promote immunothrombosis. We also discuss coronavirus disease 2019 (COVID-19)-associated ARDS as a viral comparator, the analytical limitations of redox biomarkers, and the value of serial, compartment-aware measurements. The central message is that oxidative stress is not a single systemic deficiency that can be corrected by generic antioxidants. Its biological and therapeutic significance depends on oxidant source, cellular compartment, microbial burden and disease phase. However, evidence for many specific redox pathways remains predominantly preclinical, and their clinical relevance requires further validation. At present, no validated redox-directed treatment-selection strategy exists; future approaches will therefore require validated target-engagement markers and phenotype- and time-specific trial designs.

## 1. Introduction

Acute respiratory distress syndrome (ARDS) is a clinical syndrome of acute hypoxemic respiratory failure caused by inflammatory injury to the lung and consequent non-cardiogenic pulmonary edema. The 2024 global ARDS definition retains the core requirement of acute onset, bilateral pulmonary opacities and hypoxemia, while extending recognition to patients receiving high-flow nasal oxygen and allowing the use of pulse oximetry and lung ultrasound in defined settings [1]. Despite these advances, the clinical definition of ARDS continues to be challenged by evolving patterns of respiratory support, differences in underlying risk factors and limitations in diagnostic approaches across clinical settings [2]. This broader definition is clinically useful, but it also underlines that ARDS is a syndrome rather than a single disease entity [1].

Clinically, ARDS is characterized by rapidly progressive hypoxemic respiratory failure, reduced respiratory-system compliance and bilateral pulmonary infiltrates caused by non-cardiogenic, protein-rich pulmonary edema. Many patients require invasive mechanical ventilation, which is life-saving but can aggravate lung injury when excessive tidal volumes, airway pressures or cyclic alveolar collapse impose additional mechanical stress on an already injured and heterogeneous lung. Management therefore combines prompt treatment of the underlying insult with lung-protective ventilation, appropriate positive end-expiratory pressure and, in selected patients with severe ARDS, prolonged prone positioning [3]. Despite these advances in supportive care, the underlying endothelial, epithelial and systemic inflammatory injury remains insufficiently targeted by current pharmacological therapies.

The burden of ARDS remains substantial. In the international Large Observational Study to Understand the Global Impact of Severe Acute Respiratory Failure (LUNG SAFE) prospective cohort, ARDS accounted for 10.4% of intensive-care-unit admissions and 23.4% of patients requiring mechanical ventilation; hospital mortality was 35% in mild, 40% in moderate and 46% in severe ARDS [4]. Consistent with the prognostic relevance of disease severity, the Berlin definition demonstrated a stepwise increase in mortality from mild to moderate and severe ARDS (27%, 32%, and 45%, respectively), accompanied by fewer ventilator-free days and longer durations of mechanical ventilation among survivors [5]. Despite this substantial mortality, no pharmacological therapy has demonstrated a consistent benefit across the heterogeneous ARDS population. Evidence from randomized trials nevertheless indicates that dexamethasone may improve outcomes in selected patients with established moderate-to-severe ARDS, although the evidence remains more context-dependent than that supporting low tidal volume ventilation across ARDS severity categories and early prolonged prone positioning in appropriately selected patients with moderate-to-severe ARDS [6,7,8]. For patients with very severe ARDS and refractory hypoxemia, venovenous extracorporeal membrane oxygenation (ECMO) may provide a benefit in selected patients. Its use depends on ARDS severity, timing, patient selection and institutional expertise, while early continuous neuromuscular blockade has not consistently improved outcomes [9,10]. Current clinical practice guidelines therefore make several conditional recommendations and emphasize that treatment decisions should account for individual patient and illness characteristics, reflecting the context dependence and variable certainty of the available evidence [11]. Together with treatment of the precipitating disorder and careful supportive management, these interventions remain central to current ARDS care [12,13].

This context-dependent therapeutic response is closely related to the marked heterogeneity of ARDS. ARDS may follow a direct pulmonary insult, such as bacterial or viral pneumonia, including severe acute respiratory syndrome coronavirus 2 (SARS-CoV-2) pneumonia or aspiration, or develop after extrapulmonary injury, including sepsis, pancreatitis, trauma or transfusion [13]. These conditions differ in their initial site of injury, temporal course, immune activation and contribution of endothelial and epithelial damage [13]. Direct and indirect ARDS also show distinct clinical and biological profiles, including differences in the relative prominence of epithelial injury and systemic inflammatory signals [14,15,16]. Data-driven clinical and biological subphenotypes have further shown that patients fulfilling the same syndromic definition can differ substantially in inflammatory activity, shock, coagulopathy, biomarker profiles and clinical outcome [14]. Consequently, a mechanism that is relevant in a defined subgroup may not translate into benefit when tested indiscriminately across the full ARDS population [14,17].

**Figure 1:**
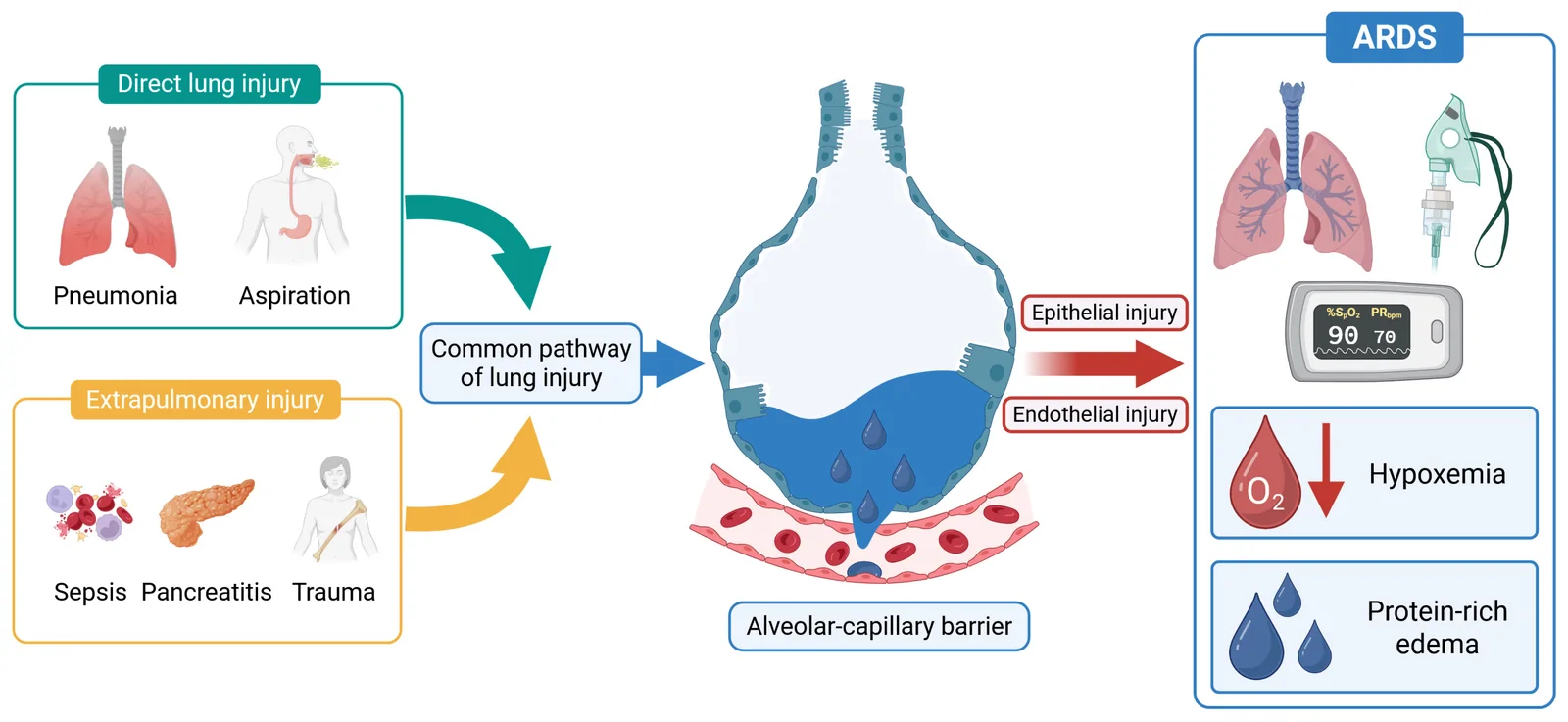
Clinical framework of acute respiratory distress syndrome. Acute respiratory distress syndrome (ARDS) can arise from direct lung injury, including pneumonia and aspiration, or from extrapulmonary insults such as sepsis, pancreatitis and trauma. Despite different initiating events, these conditions converge on injury to the alveolar–capillary barrier. Loss in endothelial and epithelial integrity increases permeability and allows protein-rich fluid to enter the alveolar space. The resulting pulmonary edema disrupts gas exchange and contributes to hypoxemia, the defining clinical manifestation of ARDS. Created in BioRender (access date 14 October 2026; Pollinger, K. (2026) https://BioRender.com/8fu4i1b).

The alveolar–capillary barrier provides a useful framework for these differences. ARDS develops when inflammatory injury increases pulmonary vascular and epithelial permeability, allowing protein-rich fluid to enter the interstitium and alveolar space. Endothelial activation, intercellular-junction disruption, leukocyte recruitment and coagulation abnormalities contribute to this process, while injury to alveolar epithelial cells compromises fluid clearance and repair [13]. Impaired alveolar fluid clearance is present in the majority of patients with acute lung injury and ARDS and is associated with longer mechanical ventilation and higher mortality, highlighting the importance of epithelial dysfunction in the resolution of pulmonary edema [18]. The relative contribution of these processes may differ between direct and indirect lung injury, making etiology clinically relevant rather than merely descriptive [17,19]. The clinical framework of direct and indirect ARDS is summarized in Figure 1.

Sepsis is one of the most important extrapulmonary triggers of ARDS and is a prototypic example of infection-driven systemic lung injury. Pathogen-associated molecular patterns (PAMPs) and host-derived damage-associated molecular patterns (DAMPs) activate pattern-recognition receptors, initiate cytokine production and alter endothelial, leukocyte and coagulation responses [20,21,22]. In sepsis, endothelial dysfunction and microcirculatory failure can occur alongside pulmonary inflammation and are linked to capillary leak, tissue hypoperfusion and organ dysfunction [21]. Clinical studies further suggest that circulating markers of endothelial injury are associated with mortality and may identify patients at an increased risk of developing ARDS after non-pulmonary sepsis, supporting a role of endothelial injury in both disease pathogenesis and biological heterogeneity [23,24]. In a prospective cohort of critically ill patients with sepsis, early plasma angiopoietin-2 was prognostic for both ARDS and mortality [24]. Circulating mitochondrial DNA may provide an additional, but still associative, signal of systemic injury: in prospective trauma and sepsis cohorts, plasma mitochondrial DNA measured approximately 48 h after presentation was independently associated with incident ARDS; in sepsis, this association was stronger for non-pulmonary than for pulmonary infectious sources [25]. A systematic review likewise found higher blood-based mitochondrial biomarkers in ARDS, while emphasizing their current investigational status [26]. Conversely, serial measurement of a soluble receptor for advanced glycation end products (sRAGE), angiopoietin-2 and surfactant protein D in sepsis-induced ARDS has not established these markers as diagnostic tools for ARDS, underscoring the need to distinguish prognostic association from validated patient selection [27].

Severe acute pancreatitis, particularly when accompanied by pancreatic or peripancreatic necrosis, represents a distinct extrapulmonary route to lung injury. Its early phase is characterized by sterile inflammation and may be complicated by persistent organ failure, whereas later deterioration can be driven by infected necrosis and secondary sepsis [28,29]. Lung injury is among the earliest and most consequential remote-organ complications of severe acute pancreatitis. Mechanistically, pancreatitis-associated acute lung injury involves diffuse alveolar and microvascular injury, inflammatory-cell recruitment and the release of proteases and pro-inflammatory mediators, with the gut and mesenteric lymphatic system also implicated as potential contributors to pulmonary injury [30]. Experimental work showed that pancreatitis-associated lung injury can involve neutrophil-dependent, oxygen-radical-mediated injury of the alveolar–capillary endothelium [31]. The contemporary clinical and mechanistic literature similarly links pancreatic cell injury, DAMP release, systemic inflammatory signaling, intestinal-barrier dysfunction and pulmonary immune-cell recruitment to acute lung injury in severe pancreatitis [28]. Preclinical evidence further suggests that impaired antioxidant defense may contribute to distant-organ injury in severe acute pancreatitis, with activation of the murine nuclear factor erythroid 2-related factor 2 (Nrf2) pathway associated with reduced oxidative stress and protection against pancreatic and pulmonary injury in experimental models [32].

Reactive oxygen species (ROS) are positioned at the intersection of these pathways. Excessive or dysregulated ROS generation can amplify ARDS-associated injury through endothelial dysfunction, lipid and protein oxidation, inflammatory signaling and regulated cell-death pathways, thereby further compromising the alveolar–capillary barrier [33]. At controlled concentrations, ROS participate in signaling, antimicrobial defense and adaptation to cellular stress; oxidative injury arises when ROS generation exceeds compartment-specific antioxidant capacity or when oxidant production becomes spatially and temporally dysregulated [34]. In acute inflammation, this imbalance can amplify endothelial activation, impair mitochondrial function, modify proteins and lipids, and influence inflammatory cell death. The extracellular redox environment also shapes immune-cell activation and tissue inflammation, emphasizing that redox regulation is not confined to intracellular metabolism [35].

Whereas recent reviews have comprehensively summarized molecular mechanisms of oxidative stress in ARDS, including NADPH oxidases (NOX) and mitochondria-derived ROS, inflammatory signaling, barrier disruption and regulated cell-death pathways [33], an important unresolved question is how these mechanisms are shaped by the initiating extrapulmonary disease process and its temporal evolution. The novelty of this review lies in integrating redox biology with disease etiology and temporal evolution, directly comparing sepsis- and pancreatitis-associated ARDS rather than considering oxidative stress as a uniform mechanism across ARDS. In this narrative review, we address this question by comparing sepsis and severe acute pancreatitis as clinically distinct, yet mechanistically convergent, drivers of ARDS. We examine how infection-driven systemic inflammation and the initially sterile inflammatory response to pancreatic necrosis engage redox-sensitive pathways of alveolar–capillary barrier failure, including the contribution of pancreas–gut–lung crosstalk and the transition to infected necrosis. We further discuss biomarkers and therapeutic approaches in relation to disease stage, biological compartment and patient heterogeneity. Our conceptual framework therefore follows the sequence from the initiating insult—either infection or initially sterile pancreatic necrosis—through the resulting PAMP- or DAMP-dominant inflammatory context and compartment-specific redox injury, to alveolar–capillary barrier failure, biomarker interpretation and the constraints on redox-directed therapy. Unless explicitly stated otherwise, mechanistic inferences in this review are based predominantly on experimental and translational studies; human studies are interpreted as associative unless prospective interventional data establish causal or treatment-selective relevance. Rather than proposing disease-specific antioxidant treatment, this comparison defines the clinical context in which a redox biomarker or intervention would need to be interpreted. COVID-19-associated ARDS is included as a viral comparator to distinguish redox-sensitive features shared across infection-associated ARDS from those that remain dependent on the initiating trigger and disease phase.

## 2. ROS, Oxidative Stress and Failure of the Alveolar–Capillary Barrier

Reactive oxygen species (ROS) are not synonymous with oxidative injury. ROS is an umbrella term that includes superoxide (O2^•−)^, hydrogen peroxide (H_2_O_2_) and more reactive oxidants generated from them. At low, locally restricted concentrations, these species act as signaling molecules in oxygen sensing, host defense and adaptive responses to cellular stress [34,36]. Oxidative stress develops when ROS production exceeds the local capacity to neutralize or repair oxidant damage, or when oxidants arise in the wrong cellular compartment. This distinction is important in ARDS: neutrophil- and mitochondria-derived ROS support antimicrobial defense, but sustained or misdirected production can damage the vascular and epithelial barriers required for gas exchange [34,37].

**Figure 2:**
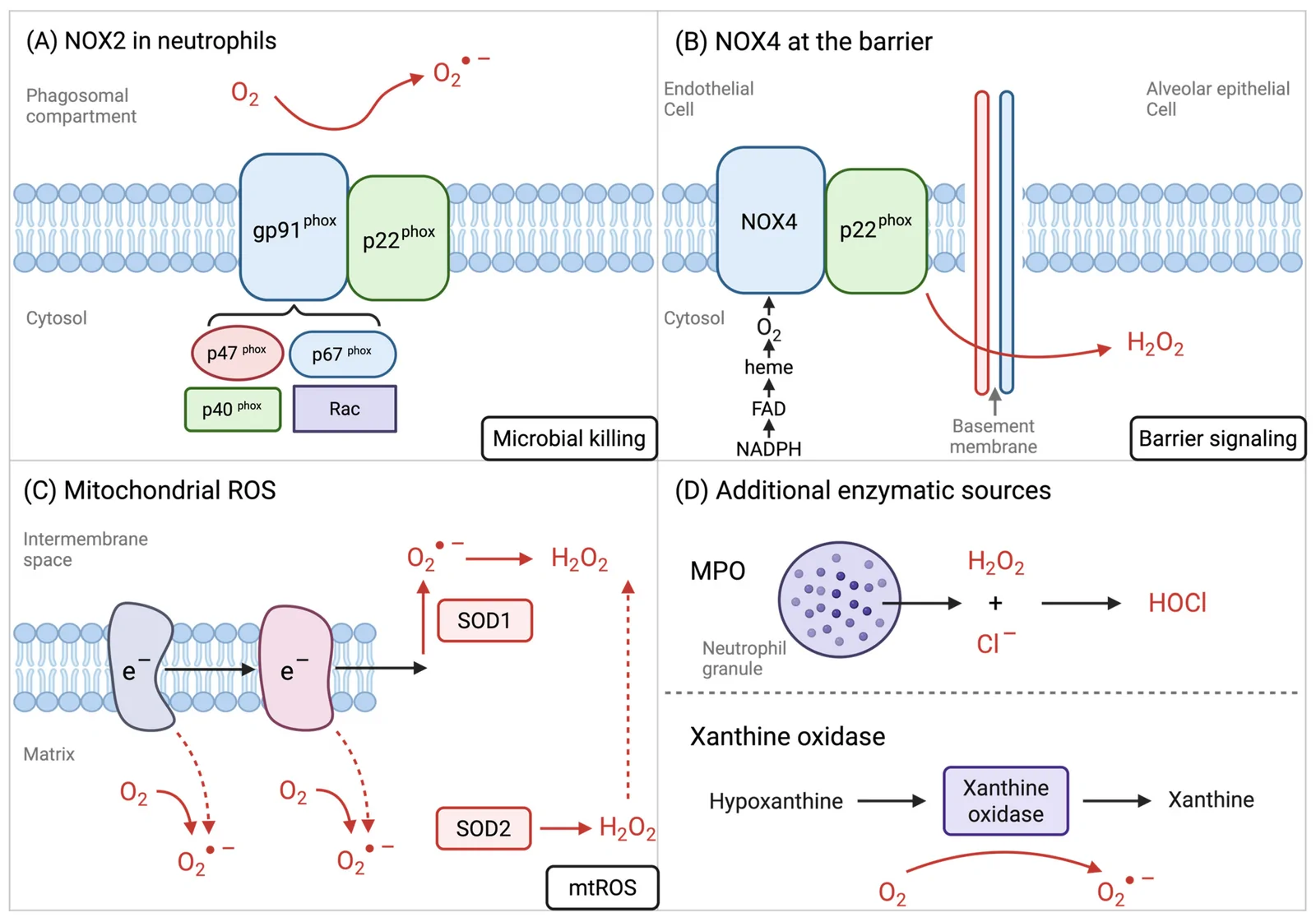
Compartmentalized oxidant generation. (**A**) Major cellular sources of reactive oxygen species (ROS) are shown as distinct mechanistic modules. In activated neutrophils, NADPH oxidase 2 (NOX2; gp91^phox^/p22^phox^) is assembled with the cytosolic regulatory subunits p47^phox^, p67^phox^, p40^phox^ and Rac family small GTPase 2 (RAC2). Electron transfer from NADPH via flavin adenine dinucleotide (FAD) and heme groups reduces oxygen to superoxide within the phagosomal compartment, thereby contributing to microbial killing. (**B**) NADPH oxidase 4 (NOX4), together with p22^phox^, is expressed in endothelial and epithelial cells and primarily generates hydrogen peroxide (H_2_O_2_), which can regulate redox-sensitive barrier signaling. (**C**) Mitochondrial ROS (mtROS) arise when electrons leak from respiratory-chain complexes I and III to molecular oxygen, generating superoxide (O_2_^•−^). Superoxide is converted to H_2_O_2_ by superoxide dismutase 1 (SOD1) in the intermembrane space and by SOD2 in the mitochondrial matrix. (**D**) Additional oxidants are produced by myeloperoxidase (MPO), which converts H_2_O_2_ and chloride (Cl^−^) to hypochlorous acid (HOCl), and by xanthine oxidase during purine metabolism. Created in BioRender (access date 14 October 2026; Pollinger, K. (2026) https://BioRender.com/m9ddbd3).

Several sources contribute to the pulmonary oxidant burden. NADPH oxidases (NOX enzymes) are dedicated ROS-generating enzymes that transfer electrons from NADPH to oxygen, thereby producing superoxide. NOX2 is abundant in phagocytes, including neutrophils, where it supplies oxidants to the phagosome for microbial killing. NOX4 is expressed in endothelial and epithelial cells and tends to generate hydrogen peroxide, linking it to redox-sensitive barrier signaling [33,36]. At the molecular level, NOX2 activation requires assembly of the gp91^phox^/p22^phox^ membrane complex with cytosolic regulatory subunits, whereas NOX4 is more constitutively active and primarily regulated by its expression level and cellular context. Accordingly, NOX2-dependent ROS are integral to phagocyte host defense, while sustained NOX4-associated signaling can promote endothelial barrier dysfunction in experimental septic lung injury [38,39] (Figure 2A,B). These isoform-specific mechanisms are supported predominantly by cellular and animal studies. In human ARDS, neutrophil activation and endothelial injury are documented, but a causal role for NOX2 or NOX4 in individual patients has not been established [23,40].

Mitochondria are a second major source: when electron transport is impaired by hypoxia, inflammation, calcium overload or defective mitochondrial quality control, electrons leak to oxygen and generate mitochondrial ROS (mtROS) (Figure 2C). Xanthine oxidase, uncoupled nitric-oxide synthase and myeloperoxidase-derived oxidants provide additional sources in inflamed tissue [36] (Figure 2D). These systems are interconnected: mitochondrial dysfunction may sustain inflammatory signaling and NOX activation, while inflammatory mediators can further impair mitochondrial respiration [33].

Reactive nitrogen species (RNS) provide a complementary redox pathway in ARDS. Inflammatory induction of inducible nitric-oxide synthase (iNOS) can increase nitric oxide (NO) production, whereas oxidative modification or cofactor deficiency may uncouple endothelial nitric-oxide synthase (eNOS), causing it to generate superoxide rather than NO. The rapid reaction of NO with superoxide produces peroxynitrite (ONOO^−^), a potent oxidant that can nitrate protein tyrosine residues, yielding 3-nitrotyrosine, and can impair mitochondrial respiration and endothelial barrier function. In experimental acute lung injury, NOX2-dependent eNOS uncoupling promoted superoxide generation and pulmonary microvascular barrier dysfunction. Reduced NO bioavailability may further disturb vasomotor regulation and microcirculatory perfusion, thereby linking oxidative and nitrosative stress to endothelial activation, permeability injury and immunothrombosis. Together, these mechanisms illustrate that redox injury arises when local ROS and RNS formation exceeds the capacity for compartment-specific buffering, repair and restoration of redox homeostasis [41,42].

**Figure 3:**
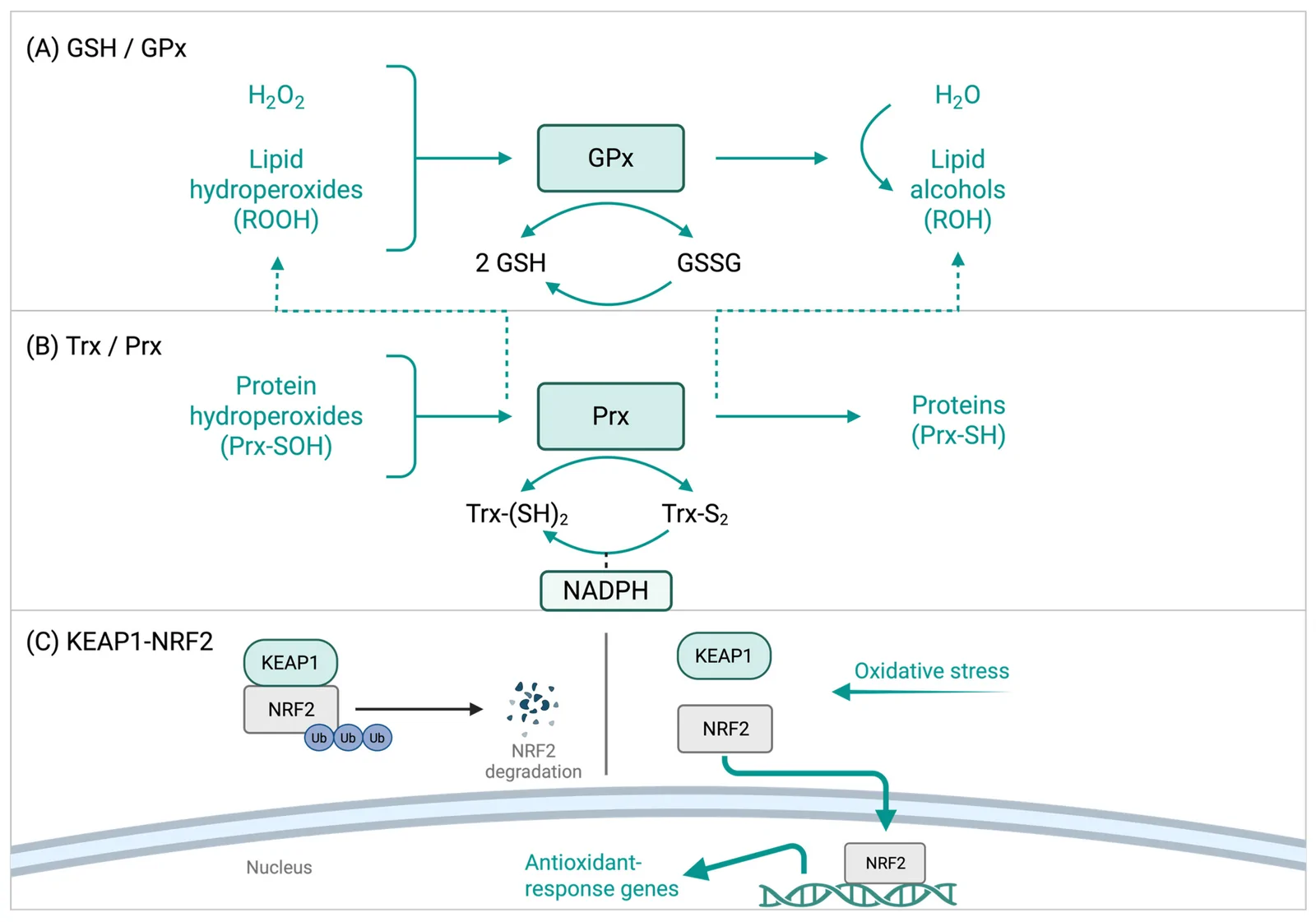
Antioxidant defense in the injured lung. (**A**) The glutathione (GSH)/glutathione peroxidase (GPx) system reduces hydrogen peroxide (H_2_O_2_) and lipid hydroperoxides at the expense of GSH, forming oxidized glutathione (GSSG). (**B**) The thioredoxin (Trx)/peroxiredoxin (Prx) system reduces protein hydroperoxides and supports redox signaling. Both systems depend on reducing equivalents ultimately derived from nicotinamide adenine dinucleotide phosphate (NADPH). (**C**) Under basal conditions, Kelch-like ECH-associated protein 1 (KEAP1) promotes ubiquitination and degradation of nuclear factor erythroid 2-related factor 2 (NRF2). Oxidative or electrophilic stress disrupts this process, allowing NRF2 stabilization, nuclear translocation and transcription of antioxidant-response genes. Created in BioRender (access date 14 October 2026; Pollinger, K. (2026) https://BioRender.com/14nk6dw).

Containment of these oxidant and nitrosative signals depends on compartmentalized antioxidant defense systems. The glutathione system uses reduced glutathione (GSH) as an electron donor; glutathione peroxidases convert hydrogen peroxide and lipid hydroperoxides into less reactive products. The thioredoxin–peroxiredoxin system reduces protein hydroperoxides and also shapes redox signaling. Both systems require reducing equivalents supplied principally by NADPH, the same cofactor used by NOX enzymes for ROS generation. Nuclear factor erythroid 2-related factor 2 (NRF2) is a stress-responsive transcription factor that increases expression of genes involved in glutathione synthesis, thioredoxin-related defense, xenobiotic metabolism and cellular redox homeostasis [37,43,44]. Under basal conditions, Kelch-like ECH-associated protein 1 (KEAP1) promotes NRF2 ubiquitination and proteasomal degradation. Redox-active and electrophilic stress modify critical KEAP1 cysteine residues, allowing NRF2 stabilization, nuclear accumulation and antioxidant-response-element-driven transcription [37] (Figure 3). These pathways should not be viewed as interchangeable antioxidants. Their effectiveness depends on the oxidant species, cellular compartment and temporal phase of injury [34,36].

In the lung, the relevant target is the alveolar–capillary barrier. Pulmonary endothelial cells regulate vascular permeability through adherent junctions, cytoskeletal organization and the endothelial glycocalyx. ROS, cytokines, bacterial products and coagulation mediators can destabilize VE-cadherin-based contacts and promote actomyosin contraction, thereby opening inter-endothelial gaps [19]. As an experimental example, NOX4-associated ROS activated a calcium/calmodulin-dependent protein kinase II (CaMKII)/extracellular signal-regulated kinase 1/2 (ERK1/2)/myosin light-chain kinase pathway, accompanied by the loss in zonula occludens-1 (ZO-1) and occludin and increased endothelial leak. This provides a defined redox-sensitive link between oxidant production, cytoskeletal contraction and junctional failure, although its relevance as a therapeutic target in human ARDS remains unresolved [39]. The resulting increase in vascular permeability contributes to protein-rich edema and impaired oxygenation. Oxidative stress also favors endothelial activation, leukocyte and platelet adhesion, and a procoagulant state, linking capillary leak to microvascular inflammation [19,45].

Alveolar epithelial injury is equally important. Type I cells form much of the gas-exchange surface, whereas type II cells contribute to surfactant production, alveolar fluid clearance and epithelial repair. By lowering surface tension at the air–liquid interface, surfactant stabilizes alveoli and helps limit alveolar flooding. In ARDS, protein-rich edema, inflammatory mediators and oxidant injury can impair surfactant composition and function, increasing surface tension, promoting alveolar derecruitment and worsening epithelial injury and alveolar fluid clearance [46,47,48]. Oxidative stress and mitochondrial dysfunction can impair these functions directly and can also influence apoptosis, pyroptosis, necroptosis and ferroptosis. Loss in epithelial integrity therefore aggravates edema not only by increasing permeability but also by limiting clearance of alveolar fluid and the capacity for regeneration [12,49]. The current evidence for many epithelial redox mechanisms is derived from experimental systems; their relative contribution in individual patients with ARDS remains to be established [12,49].

Mechanical ventilation and oxygen therapy can further modify the pulmonary redox environment. These treatment-related modifiers are not specific to either sepsis-associated or severe acute pancreatitis-associated ARDS; they may amplify pre-existing epithelial and endothelial injury across ARDS etiologies. In experimental systems, excessive cyclic stretch and hyperoxia increase ROS generation, promote mitochondrial dysfunction and NADPH oxidase activation, and aggravate epithelial and endothelial barrier injury. These observations provide a mechanistic rationale for lung-protective ventilation and avoidance of unnecessary hyperoxia in ARDS, although they do not establish a redox-directed treatment strategy [50,51,52,53,54].

Neutrophils provide a mechanistic bridge between oxidant production, tissue injury and immunothrombosis. During activation, NOX2-dependent oxidant generation contributes to microbial killing and can facilitate neutrophil extracellular trap (NET) formation. NETs are extracellular webs of DNA decorated with histones and antimicrobial proteins. They can immobilize microbes, but excessive NET accumulation exposes the lung microvasculature and alveolar compartment to histones, proteases, oxidants and extracellular DNA, promoting endothelial injury, epithelial damage and coagulation [55,56]. These observations establish a plausible link between oxidant signaling, NET formation and barrier injury, but they do not yet justify NET-directed treatment in clinical ARDS.

Inflammasomes add another redox-sensitive layer. Inflammasomes are cytosolic innate-immune signaling complexes; the NOD-like receptor family pyrin domain-containing 3 (NLRP3) inflammasome is the best-studied example in ARDS. Oxidative stress, mitochondrial injury and DAMP release can promote its activation, leading to caspase-1-dependent processing of interleukin (IL)-1β and IL-18 and, in appropriate cellular contexts, gasdermin-dependent pyroptosis. Pyroptosis is an inflammatory form of lytic cell death that can release further damage-associated molecular patterns (DAMPs) [56]. Inflammasome signaling is therefore positioned to amplify barrier injury and to connect innate immune sensing with inflammatory cell death [57]. However, the direction and magnitude of this contribution vary with the insult, cell type and disease stage. Broad inhibition of inflammatory pathways without attention to these variables risks compromising microbial clearance or repair [57].

Lipid peroxidation and ferroptosis deserve particular attention because they provide a direct link between redox imbalance and membrane failure. Lipid peroxidation refers to the oxidation of polyunsaturated membrane lipids; if it becomes extensive, membrane structure and function are lost. Ferroptosis is a regulated, iron-dependent form of cell death driven by the accumulation of lipid hydroperoxides. Glutathione peroxidase 4 (GPX4) is central to the protective system because it uses GSH to reduce lipid hydroperoxides before they propagate membrane damage [56,58]. Experimental and review data suggest that the ferroptotic injury of alveolar epithelial and endothelial cells may contribute to ARDS-associated barrier dysfunction. Nevertheless, this field remains largely preclinical, and ferroptosis markers are not yet validated for routine patient stratification or treatment selection in ARDS [33,56,58].

Taken together, ROS-mediated injury in ARDS should be understood as a failure of regulated redox signaling rather than as the simple presence of oxidants. NOX enzymes, mitochondria and activated neutrophils generate ROS within an immune response that also depends on ROS for host defense. The clinical challenge is to identify when and where oxidant production becomes injurious, and which defense systems have failed. Although experimental studies define many of the individual cellular pathways discussed above, clinical evidence currently supports redox-related markers mainly as indicators of injury or biological heterogeneity rather than as validated treatment-selection tools [33,34]. Accordingly, the mechanistic pathways discussed in this section should be regarded as biologically plausible frameworks, not as individually validated causal targets in human ARDS. The following sections apply this common framework to the distinct initiating contexts of infection-driven sepsis and initially sterile pancreatic necrosis, rather than re-describing the shared mechanisms.

## 3. Sepsis-Associated ARDS: Infection-Driven Oxidative Injury

According to Sepsis-3, sepsis is defined as life-threatening organ dysfunction caused by a dysregulated host response to infection [59]. Earlier consensus definitions are not used as diagnostic criteria in this review. The sepsis-related Sequential Organ Failure Assessment (SOFA) score quantifies dysfunction across respiratory, cardiovascular, hepatic, coagulation, renal and neurological organ systems [59]. Because calculation of the full SOFA score requires laboratory and clinical variables, the bedside quick SOFA (qSOFA) score may support rapid risk assessment in patients with suspected infection. qSOFA comprises systolic blood pressure ≤ 100 mmHg, altered mental status and respiratory rate ≥22/min; the respiratory component of the SOFA score primarily reflects impaired gas exchange through the ratio of arterial oxygen tension to inspired oxygen fraction (PaO_2_/FiO_2_) [59,60]. Sepsis-induced organ dysfunction is defined by an acute SOFA increase of ≥2 points attributable to infection, a threshold associated with an in-hospital mortality of approximately 10% [59]. ARDS may develop after pulmonary infection or as an indirect consequence of an extrapulmonary focus. This distinction is clinically useful but mechanistically incomplete: in both settings, circulating microbial products, inflammatory mediators, activated leukocytes and injured endothelium converge on the alveolar–capillary barrier [20]. A central hallmark of this shared pathway is redox imbalance, with increased generation of ROS and RNS by activated leukocytes, endothelial and epithelial cells, and dysfunctional mitochondria [61]. Excessive or sustained oxidant signaling can promote endothelial and epithelial barrier dysfunction, lipid and protein oxidation, and amplification of inflammatory signaling, creating a feed-forward loop between oxidative injury and inflammation [62]. The risk and phenotype of lung injury vary with infection source and pathogen type, the timing and adequacy of antimicrobial treatment and source control, disease phase, pre-existing pulmonary disease, age, host immune status and supportive care. A single redox signature should therefore not be expected across septic ARDS [33].

At the onset of infection, pathogen-associated molecular patterns (PAMPs) are detected by pattern-recognition receptors (PRRs), including Toll-like receptors, NOD-like receptors and C-type lectin receptors. Engagement of these systems activates transcriptional and post-translational programs that support cytokine production, phagocyte recruitment, complement activation and antimicrobial effector functions [20]. These innate immune pathways are tightly coupled to redox signaling. PRR engagement induces ROS and RNS generation through sources such as NOX, the mitochondrial electron transport chain and inducible nitric-oxide synthase (iNOS), while ROS can in turn modulate downstream inflammatory signaling, including Nuclear factor kappa-light-chain-enhancer of activated B cells (NF-κB) and inflammasome activation. Thus, redox perturbation is not merely a consequence of inflammation but can function as an amplifier of PAMP- and DAMP-driven signaling [63,64]. In parallel, damaged host cells release DAMPs, including high-mobility group box 1 (HMGB1), extracellular ATP and mitochondrial constituents. Thus, infection and tissue injury do not constitute separate inflammatory inputs in sepsis; they reinforce one another and can perpetuate inflammation after the initial microbial stimulus has been reduced [20,65].

In sepsis, the oxidant sources and defense systems outlined in Section 2 are activated in the setting of ongoing infection, variable pathogen burden and systemic hypoperfusion. Their pathological relevance therefore depends on whether antimicrobial oxidant functions are preserved while sustained redox dysregulation promotes lung injury [20,21,33,66].

Mitochondrial injury provides a particularly plausible bridge between cell stress and systemic inflammation. Mitochondrial DNA, formyl peptides and other mitochondrial DAMPs can enter the extracellular compartment after cell injury and activate leukocytes and endothelial cells. At the same time, mitochondrial dysfunction can increase the generation of mtROS, which may further promote oxidative damage, mitochondrial membrane dysfunction and release of mitochondrial constituents. This creates a potential feed-forward loop in which mitochondrial injury promotes both redox stress and DAMP release, while DAMP-driven inflammation and leukocyte activation can further compromise mitochondrial function [67]. Human data from patients with septic shock, rather than specifically sepsis-associated ARDS, support the clinical relevance of this bioenergetic link. In skeletal-muscle biopsies from patients with septic shock, increased nitric-oxide production and reduced GSH concentrations were associated with lower respiratory-chain complex-I activity, reduced ATP concentrations and adverse outcome [68]. In experimental systems, mitochondrial DAMPs increased endothelial permeability through an early neutrophil-independent phase followed by a sustained neutrophil-dependent phase. Inhibition of endosomal Toll-like receptor signaling attenuated these effects [65]. These experimental findings identify a plausible mechanism for vascular leak but do not establish that circulating mtDNA or other mitochondrial DAMPs are causal mediators of barrier failure in individual patients with septic ARDS.

The endothelium is both a target and an amplifier of septic inflammation. Cytokines, bacterial products, oxidants and coagulation mediators disrupt adherent junctions, the glycocalyx and cytoskeletal homeostasis, thereby promoting capillary leakage. Endothelial activation also increases leukocyte and platelet adhesion and favors a procoagulant surface, so permeability injury and microvascular thrombosis progress together rather than as independent processes. In sepsis, impaired microvascular perfusion can further aggravate regional hypoxia and mitochondrial dysfunction, thereby sustaining oxidative stress and creating a self-reinforcing cycle of endothelial dysfunction and tissue hypoxia [19,21].

Neutrophils are central to this feed-forward response. In addition to oxidant release and degranulation, activated neutrophils can form NETs. A prospective whole-blood transcriptomic study found increased expression of neutrophil-related genes in early sepsis-induced ARDS compared with sepsis without ARDS, supporting the clinical relevance of early neutrophil activation [40]. Experimental studies link NET formation to neutrophil redox signaling, with ROS, including NOX2-derived ROS, acting as signals in the transition toward NETosis [56,69]. In human sepsis-associated ARDS, these pathways remain biologically plausible but are not established as isoform-specific causal mechanisms. NET components, including histones, proteases, myeloperoxidase and DNA, may injure endothelial and epithelial cells and provide a scaffold for immunothrombosis [70,71]. In septic ARDS, tissue factor-enriched NETs were increased in patients and experimental models; interventions targeting NETs, tissue factor or thrombin reduced lung injury in mice [71]. These findings strengthen the case for NET-mediated injury, yet they do not justify indiscriminate NET suppression, because NET degradation can impair bacterial control unless infection is adequately treated [70].

Clinical and experimental observations also link neutrophil redox metabolism to disease severity. Mechanistically, aldehyde dehydrogenase 2 (ALDH2) links mitochondrial aldehyde metabolism to redox homeostasis by detoxifying reactive aldehydes generated during lipid peroxidation. Impaired ALDH2 activity may consequently increase aldehyde-mediated cellular stress and alter neutrophil signaling toward excessive NET formation and tissue injury [72]. In a recent study, an ALDH2 risk variant was associated with higher-circulating NET markers in septic ARDS, while the corresponding mouse models showed increased NETosis, vascular leakage and lung injury [72]. These findings link a patient-associated ALDH2 variant to NET-associated vascular injury in septic ARDS, but they do not establish ALDH2 status as a treatment-selection biomarker. Current human evidence in sepsis-associated ARDS is strongest for associations of neutrophil activation, NET-related markers and endothelial injury with disease severity [23,64,66,67]. By contrast, the contributions of individual NOX isoforms, mtROS or mitochondrial DAMPs, and ALDH2-dependent NETosis to alveolar–capillary barrier failure remain mechanistic hypotheses supported chiefly by cellular and animal studies [33,39,65,72]. The therapeutic implications of this context dependence, including the limitations of nonselective antioxidant approaches, are addressed in Section 5.

COVID-19-associated ARDS is included as a viral comparator, not as a universal model for ARDS. Clinical and autopsy studies demonstrate pulmonary endothelial injury, microangiopathy and NET-associated thromboinflammation [73,74]. It illustrates that a defined infectious trigger can generate temporally heterogeneous endothelial, epithelial and thromboinflammatory injury, supporting compartment- and stage-specific redox measurements [33,75]. Thus, sepsis-associated ARDS is shaped from the outset by pathogen burden and the need to preserve antimicrobial oxidant functions, whereas severe acute pancreatitis initially represents a predominantly sterile inflammatory context in which necrosis, metabolic failure and DAMP release precede, but may later converge with, infection-driven biology [28,59]. 

## 4. Severe Acute Pancreatitis-Associated ARDS: Sterile Inflammation and Pancreas–Gut–Lung Crosstalk

According to the revised Atlanta classification, interstitial edematous pancreatitis is characterized by acute pancreatic inflammation without necrosis, whereas necrotizing pancreatitis denotes pancreatic and/or peripancreatic necrosis. Clinical severity is defined independently: moderately severe acute pancreatitis involves transient organ failure (<48 h) and local or systemic complications without persistent organ failure, whereas severe acute pancreatitis is defined by persistent organ failure (>48 h) [76]. Necrotizing pancreatitis is a morphological subtype characterized by pancreatic and/or peripancreatic necrosis. This morphology does not, by itself, establish severe disease or ARDS [76]. In this review, pancreatitis-associated ARDS refers to clinically defined ARDS in patients with acute pancreatitis, whereas findings from animal and cellular models are described as experimental pancreatitis-associated acute lung injury [5]. Lung injury is a major early systemic complication of severe disease. In a 2026 meta-analysis of 17 studies including 3576 patients with severe acute pancreatitis, the pooled ARDS incidence was 36.4% (95% confidence interval [CI], 30.7–42.5%); however, substantial heterogeneity cautions against interpreting this estimate as a universal epidemiological rate [77]. In a retrospective intensive care unit (ICU) cohort of 151 patients with acute pancreatitis-related ARDS, 90-day mortality was 37%, underscoring the clinical importance of established ARDS in this setting [78]. In contrast to infection-driven ARDS, the initial inflammatory signal in acute pancreatitis, particularly when associated with pancreatic necrosis, is often sterile, arising from acinar-cell injury, premature enzyme activation, necrosis and release of endogenous danger signals [28]. The clinical relevance of necrosis lies not simply in its morphology, but in its association with a sustained sterile inflammatory burden and the potential transition to infected necrosis, superimposed sepsis and persistent organ failure.

In cellular and animal models of acute pancreatitis, sustained cytosolic calcium elevations in acinar cells can overload mitochondria, compromise oxidative phosphorylation and reduce ATP availability. Mitochondrial permeability transition, loss in membrane potential and impaired calcium extrusion then favor necrotic rather than orderly apoptotic cell death [79,80,81]. Because mitochondrial injury also promotes ROS generation, calcium overload, energy failure and oxidative stress form a mutually reinforcing process rather than a simple linear sequence [79,80,81]. In experimental acute pancreatitis, inhibition of mitochondrial permeability transition preserved acinar-cell ATP production and attenuated disease severity, supporting mitochondrial injury as an early mechanistic node in animal models. Its causal contribution to human severe acute pancreatitis-associated ARDS remains to be established [82].

In pancreatic acinar cells, sustained ROS accumulation can amplify mitochondrial dysfunction, lipid and protein oxidation, inflammatory signaling and necrosis [80,83]. Lipid peroxidation is of particular interest because it directly couples oxidant injury to membrane damage. Cellular and animal studies have linked lipid peroxidation and ferroptosis-like processes to pancreatic injury. However, the specific contribution of ferroptosis to human severe acute pancreatitis and clinically defined pancreatitis-associated ARDS remains unproven [83,84].

As outlined in Section 2, experimental cellular and animal studies indicate that mitochondrial injury and DAMP release can activate inflammasome signaling. In necrotizing pancreatitis inflammasome signaling may be initiated by acinar-cell injury and tissue necrosis: extracellular ATP, HMGB1, mitochondrial DNA and other intracellular constituents can amplify sterile inflammation, while mitochondrial ROS can promote NLRP3 activation and IL-1β/IL-18-associated responses [28,83,85]. This pathway may contribute to neutrophil recruitment, endothelial activation and inflammatory cell death, although its relative importance likely varies with disease stage and cellular context. In mice with acute pancreatitis, NLRP3 inhibition reduced both the systemic inflammatory response and the compensatory anti-inflammatory response, supporting a stage-dependent role for this pathway in experimental acute pancreatitis [86]. Whether NLRP3 activation independently contributes to pulmonary injury or identifies ARDS in patients with severe acute pancreatitis remains unknown. The relative contribution of these mechanisms is likely to change across sterile necrosis, systemic inflammatory response syndrome and later infection.

The transition from local pancreatic injury to remote pulmonary damage is thought to involve the systemic dissemination of DAMPs released from necrotic pancreatic tissue, inflammatory mediators, activated leukocytes, oxidized lipids and pancreatic products. Experimental studies suggest that the mesenteric and retroperitoneal lymphatic systems may provide additional conduits through which cytokines, pancreatic enzymes and other injurious signals reach the pulmonary circulation [87,88]. At the pulmonary microvasculature, these circulating factors may promote endothelial activation, glycocalyx injury, leukocyte adhesion and increased permeability, providing a mechanistic basis for protein-rich alveolar edema [28,89]. These mechanisms overlap substantially with indirect ARDS after sepsis, but their initiating context differs: in early experimental pancreatitis-associated lung injury, endogenous tissue injury predominates before a microbiologically documented infection is present.

Neutrophils provide a major cellular link between pancreatic inflammation and remote pulmonary injury. Systemic inflammatory signals generated during severe acute pancreatitis promote neutrophil activation, pulmonary sequestration and transendothelial migration, where neutrophil-derived ROS and proteases contribute to endothelial and alveolar injury [30,90]. As outlined in Section 2, neutrophil ROS and NET formation can link inflammatory activation to barrier injury and immunothrombosis. In pancreatitis-associated lung injury, this common effector program is driven by systemic signals originating from the injured pancreas and the evolving gut–lung axis [28,30,31,42,89,90,91]. Experimental studies suggest that neutrophil-derived ROS and proteases contribute to endothelial damage and impaired gas exchange, while NET formation contributes to the severity of acute pancreatitis and associated organ injury [90,92,93]. In observational studies of acute pancreatitis, increased NET-related markers have been associated with disease severity, but these associations do not establish NETs as causal mediators of human pancreatitis-associated ARDS [92]. However, most evidence linking NETs and immunothrombosis to pancreatitis-associated lung injury remains experimental. NETs are also components of antimicrobial host defense, and indiscriminate inhibition may therefore interfere with pathogen control and tissue repair. The therapeutic relevance of this pathway will likely depend on disease stage, the balance between sterile and infectious inflammation, and the predominant cellular compartment driving tissue injury.

The intestine is an important intermediate organ in this syndrome. Severe acute pancreatitis is associated with intestinal hypoperfusion, edema, altered motility, mucosal immune dysregulation and disruption of epithelial tight junctions. Oxidative stress and local inflammatory mediators can exacerbate this barrier failure, allowing microbial products and bacteria to access mesenteric lymphatics and the portal or systemic circulation [89,94]. Gut microbial metabolites, including short-chain fatty acids, may influence epithelial barrier function and immune regulation, but most intervention data in pancreatitis-associated lung injury remain preclinical [95]. Human studies support an association between severe acute pancreatitis, intestinal-barrier dysfunction and adverse clinical outcomes, but they do not establish a causal gut-to-lung mechanism in ARDS.

Barrier dysfunction also helps to explain the clinical transition from sterile necrosis to infected necrosis and sepsis. In a clinical and experimental study of acute necrotizing pancreatitis, bacterial translocation associated with infected pancreatic necrosis was traced predominantly to the small bowel rather than the colon [96]. This finding supports a plausible small-bowel route of infection but cannot establish bacterial translocation in individual patients or identify a universal route to infected necrosis. Once infection of necrotic collections develops, PAMP-driven sepsis mechanisms are superimposed on the pre-existing DAMP- and ROS-rich inflammatory state; the distinction between pancreatitis-associated and sepsis-associated ARDS then becomes biologically less sharp [28,87,96].

This temporal evolution has practical consequences for a redox-centered review. Oxidative stress markers and impaired antioxidant capacity correlate with disease severity in several acute pancreatitis studies, but they are not validated as treatment-selection tools for pancreatitis-associated ARDS [83,84]. Trials of broadly acting antioxidants have been heterogeneous in agent, timing and clinical endpoint. During early sterile injury, clinical priorities are organ-failure assessment and supportive care; prophylactic antibiotic therapy is not recommended for sterile necrotizing pancreatitis. Suspected or confirmed infected necrosis, however, shifts management toward antimicrobial therapy and source control [28,97]. Future studies should determine whether defined redox pathways identify a subgroup in whom pancreas-, gut- or lung-directed treatment can be tested rationally. Compared with sepsis-associated ARDS, the principal distinction is therefore temporal rather than the presence or absence of shared pulmonary injury mechanisms: sterile pancreatic injury and DAMP release predominate early, whereas infected necrosis can subsequently introduce a PAMP-driven septic component. Table 1 summarizes this comparison across initiating insult, redox-active processes, barrier injury, biomarkers and therapeutic implications.

## 5. Shared Redox Programs, Biomarkers and Therapeutic Implications

Sepsis-associated and severe acute pancreatitis-associated ARDS arise from different initiating events but converge on a limited set of injury programs. Both conditions combine endothelial activation, epithelial dysfunction, neutrophil recruitment, coagulation activation and impaired alveolar fluid clearance. Reactive oxygen species (ROS) are integrated into each program rather than representing an isolated late consequence of inflammation. NOX-derived oxidants, mtROS and myeloperoxidase-derived hypochlorous acid can alter junctional proteins and membrane lipids, while also amplifying cytokine signaling and regulated cell death [19,33,34]. The same oxidant system can therefore be beneficial or harmful according to its source, concentration, compartment and the prevailing microbial burden [19,34]. Beyond their mechanistic role, ROS and their downstream oxidation products may provide measurable signatures of the dominant injury process. However, no individual analyte can quantify “oxidative stress in the lung”; its interpretation depends on the molecular event measured, the sampled compartment, the timing of collection and the underlying disease trajectory.

The comparison in Table 1 provides the clinical framework for the shared redox programs discussed below: the same molecular pathway may have different biological meaning and therapeutic implications according to initiating insult, microbial burden, compartment and disease phase. In both settings, the pulmonary endothelium is exposed to circulating cytokines, complement products, extracellular histones, mitochondrial DAMPs and procoagulant mediators. It translates these inputs into permeability, leukocyte adhesion and immunothrombosis [19,35]. This model explains why indirect ARDS following sepsis and pancreatitis may have related physiology but differ in inflammatory composition, temporal dynamics and response to treatment.

**Figure 4:**
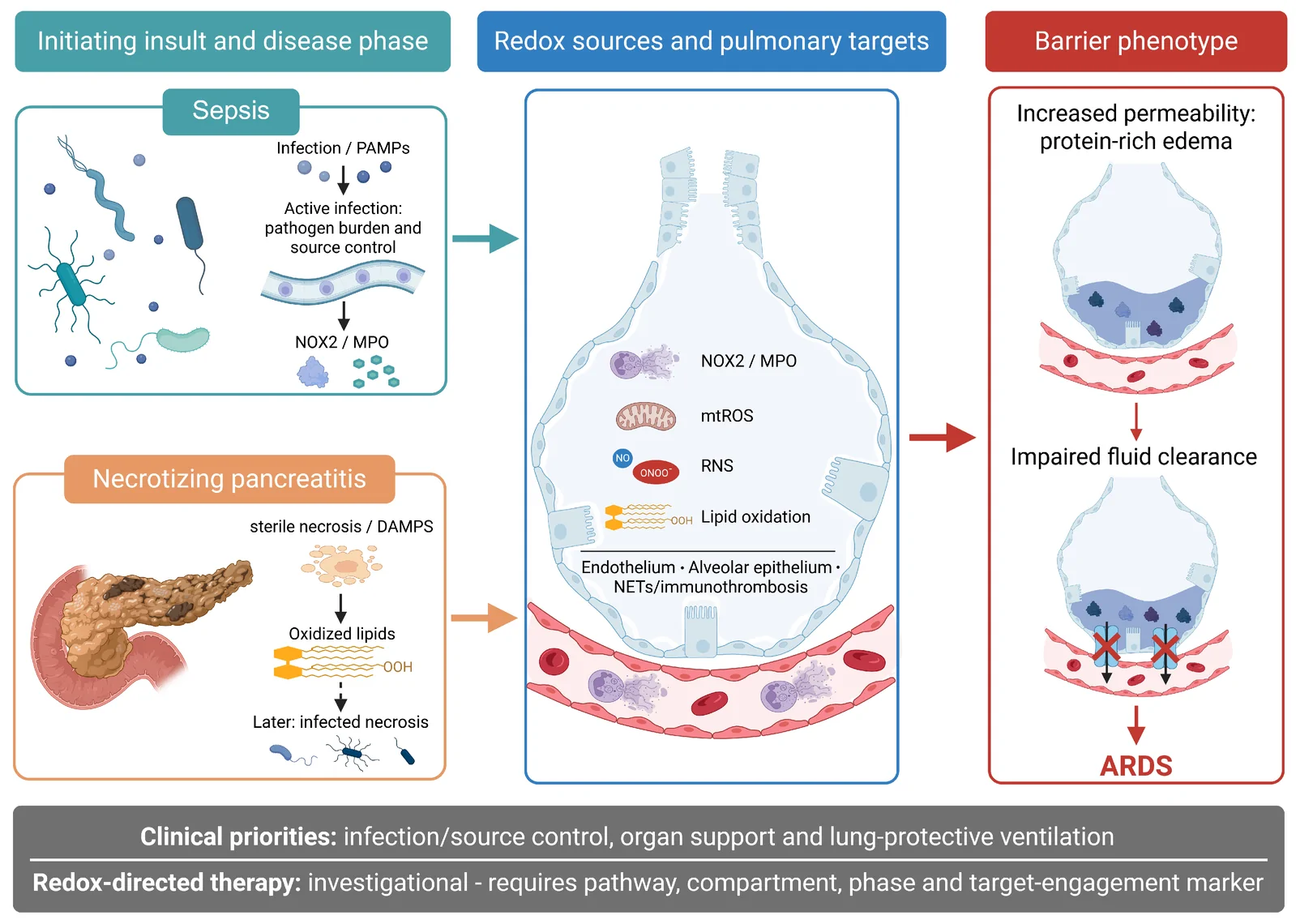
From initiating insult to phase-specific translational implications in indirect acute respiratory distress syndrome (ARDS). Sepsis and severe acute pancreatitis initiate distinct inflammatory contexts. Infection generates a PAMP-dominant response in which pathogen burden, antimicrobial host defense and source control are central, whereas early severe acute pancreatitis is characterized by sterile acinar-cell injury, damage-associated molecular pattern (DAMP) release, metabolic stress and oxidized lipids; infected necrosis may subsequently add a pathogen-associated molecular pattern (PAMP)-driven septic component. These triggers engage compartment-specific redox sources, including NADPH oxidase 2 (NOX2)/myeloperoxidase-derived oxidants, mitochondrial reactive oxygen species, reactive nitrogen species and lipid oxidation. At the alveolar–capillary barrier, endothelial activation, epithelial dysfunction and neutrophil/neutrophil extracellular trap (NET)-associated immunothrombosis converge on increased permeability, protein-rich edema and impaired alveolar fluid clearance. The resulting ARDS phenotype therefore requires phase-specific clinical management. Redox-directed interventions remain investigational and would require a defined pathway, relevant compartment, treatment window and target-engagement marker. Created in BioRender (access date 14 October 2026; Pollinger, K. (2026) https://BioRender.com/bu7io8o).

The term immunothrombosis is useful because it emphasizes that coagulation is part of the host defense but can become maladaptive. Endothelial tissue-factor expression, platelet activation, NET scaffolds, complement activation and impaired fibrinolysis promote fibrin deposition in the pulmonary microcirculation [70,71]. Oxidative stress can intensify this response through endothelial activation, nitric-oxide dysregulation and lipid oxidation [19]. The distinct initiating events and their convergence on redox-sensitive alveolar–capillary barrier failure are summarized in Figure 4.

No circulating measurement can quantify “oxidative stress in the lung” as a single entity. For clarity, biomarker interpretation should distinguish four non-equivalent categories: markers of oxidant sources or generation; direct molecular footprints of oxidation; measures of antioxidant capacity or redox defense; and markers of downstream endothelial or epithelial tissue injury. Biomarkers validated for prognostic or treatment-selection purposes constitute a separate level of clinical validation. Biomarkers should instead be interpreted according to the biological compartment and process they most closely reflect. Angiopoietin-2 (Ang-2) indicates perturbation of the endothelial angiopoietin signaling axis; soluble thrombomodulin and syndecan-1 reflect endothelial injury and glycocalyx shedding; and soluble receptor for advanced glycation end products (sRAGE), surfactant protein D (SP-D), club cell secretory protein 16 (CC16) and Krebs von den Lungen-6 (KL-6) have been used as markers of epithelial injury rather than as specific measures of oxidative stress [100,101,102]. These markers reflect tissue response or barrier injury [103]; they neither quantify ROS generation nor establish oxidative stress as the dominant mechanism of injury. Ang-2 has shown a diagnostic and prognostic signal in meta-analyses, but substantial between-study heterogeneity precludes use of a universal decision threshold [100]. Routine critical-care measurements, including the PaO_2_/FiO_2_ ratio, platelet count, D-dimer, C-reactive protein and standard organ-function tests, are readily available and can support clinical assessment and risk stratification. However, they are indirect measures of inflammation, coagulation or organ injury rather than specific measures of pulmonary redox biology. In contrast, Ang-2, sRAGE, SP-D, NET-related markers and circulating mtDNA remain predominantly investigational biomarkers in this setting.

Inflammatory and thromboinflammatory markers answer complementary, not identical, questions. Interleukin (IL)-6, IL-8, soluble tumor-necrosis-factor receptor-1, ferritin and plasma bicarbonate have contributed to the classification of hyperinflammatory ARDS, whereas cell-free DNA, myeloperoxidase–DNA complexes and citrullinated histones are commonly used as indirect measures of NET formation [91,104]. These are surrogate markers that may also reflect cell death or neutrophil activation and do not specifically or quantitatively establish in vivo NET formation. Circulating mitochondrial DNA may identify a DAMP-rich state; in septic cohorts, higher plasma mitochondrial-DNA concentrations have been associated with acute lung injury and mortality [98]. These associations support enrichment strategies, but they do not prove that the measured molecule is the principal causal mediator in an individual patient. NET-related markers and circulating mtDNA should likewise not be interpreted as direct measures of ROS generation. They reflect neutrophil activation or mitochondrial injury, respectively. Markers associated with prognosis or biological heterogeneity should therefore be distinguished from biomarkers validated for treatment selection. At present, no redox-related, endothelial, epithelial or NET-associated marker is validated to select patients for redox-directed therapy. A target-engagement marker would additionally need to show that a defined intervention has altered its intended pathway in the relevant compartment; this remains a prospective research requirement.

Redox-oriented measurements require particular caution. Myeloperoxidase (MPO) can provide source-related information on neutrophil oxidant activity, whereas the ratio of reduced to oxidized glutathione (GSH/GSSG) reflects redox-buffering capacity. Circulating mitochondrial DNA is better interpreted as a marker of mitochondrial DAMP release than as a direct measure of oxidant generation. F_2_-isoprostanes, malondialdehyde and 4-hydroxynonenal adducts are molecular footprints of lipid oxidation, whereas oxidized glutathione and the glutathione redox ratio reflect thiol redox balance. None of these measurements directly identifies the rate or cellular source of ROS generation. Their analytical behavior differs substantially between plasma, bronchoalveolar lavage fluid and tissue. In ARDS, bronchoalveolar lavage studies have identified increased oxidized glutathione and F2-isoprostanes, consistent with an oxidant burden in the alveolar compartment [99,105,106,107]. Yet a high-circulating lipid-peroxidation signal may arise from liver dysfunction, renal failure, transfusion, hemolysis or systemic inflammation, rather than from the injured lung alone. Consistent with this limitation, in severe sepsis, plasma F2-isoprostanes and isofurans were associated with renal, hepatic and coagulation failure, but not pulmonary failure [108]. Pre-analytical handling, sample timing and the assay platform must therefore be specified before a redox biomarker can be used for patient selection. This requirement has practical analytical consequences: F2-isoprostanes should preferentially be quantified by validated mass-spectrometric methods. Although analytically robust, such assays are not currently available with standardized, rapid turnaround in most intensive-care settings and should therefore be regarded as research tools rather than near-term tests for clinical stratification. Malondialdehyde (MDA) and thiobarbituric acid-reactive substance (TBARS) assays have limited analytical specificity because thiobarbituric acid reacts with multiple aldehydes and other sample constituents; they should therefore not be interpreted as standalone measures of lipid peroxidation. Measurement of the GSH/GSSG ratio requires immediate thiol stabilization and standardized sample processing to prevent ex vivo oxidation artifacts [109,110,111].

Interpretation must also respect the sampled biological compartment. Plasma is readily available but integrates systemic inflammation, organ dysfunction and treatment effects and therefore cannot be assumed to represent lung-specific redox biology. Bronchoalveolar lavage fluid samples the alveolar compartment more directly, but lavage dilution, regional sampling variability and limited feasibility in critically ill patients constrain interpretation. Circulating blood cells allow cell-resolved assessment of leukocyte activation and redox-related transcriptional programs, but do not necessarily report endothelial or epithelial processes in the lung. Lung tissue provides the closest spatial information, yet human samples are usually restricted to biopsy or post-mortem material and may be affected by the sampling bias. Exhaled breath and exhaled-breath condensate offer non-invasive access to volatile and non-volatile airway-derived signals, but analytical standardization and their relationship to distal alveolar injury remain insufficiently established [105,112,113,114].

**Figure 5:**
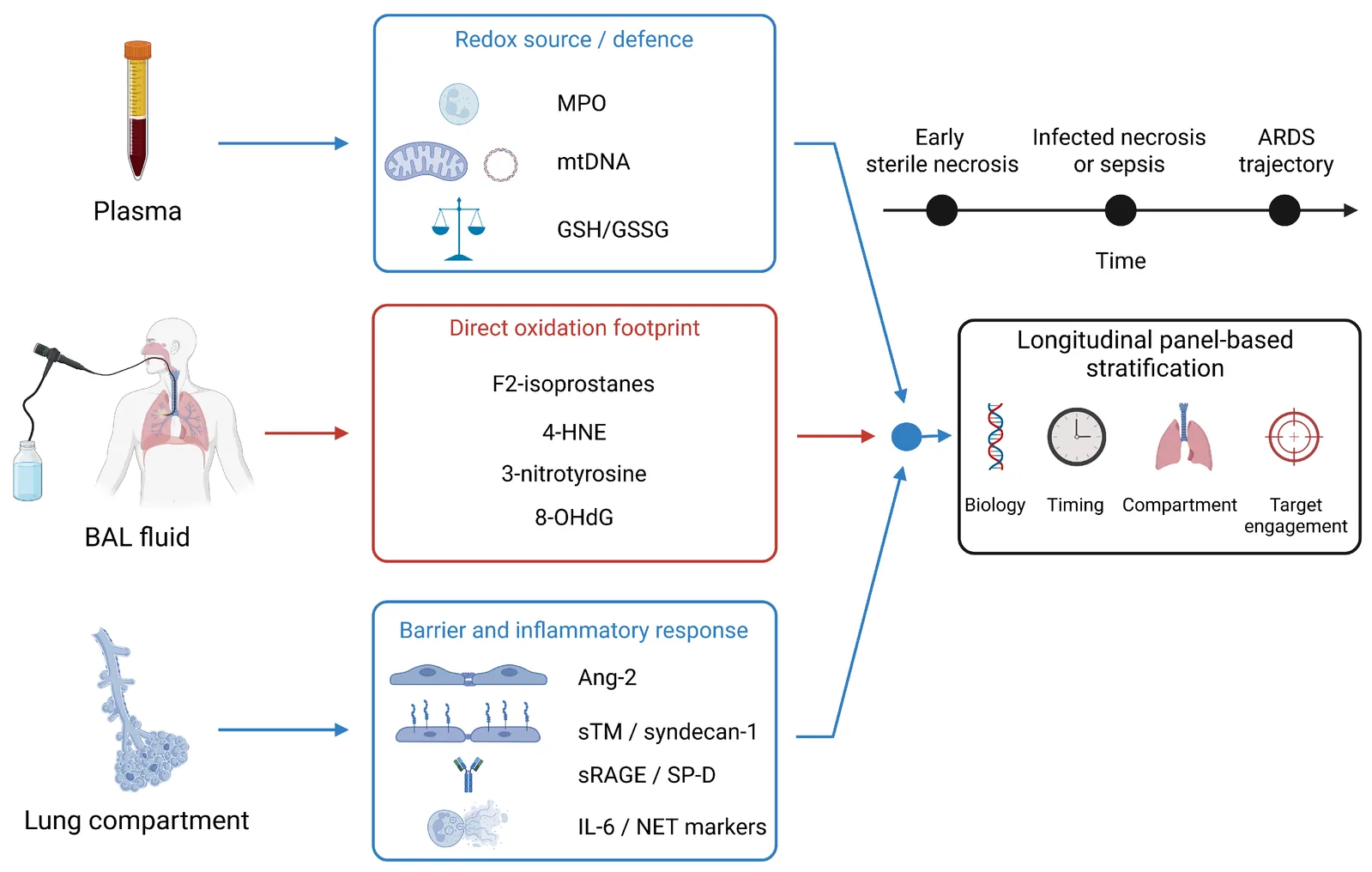
Redox biology in indirect acute respiratory distress syndrome (ARDS) cannot be captured by a single circulating marker. Plasma, bronchoalveolar lavage (BAL) fluid and lung-derived samples provide complementary, but non-equivalent, information and should be interpreted in relation to disease stage. A biomarker panel may include process-related markers, such as myeloperoxidase (MPO), which reflects neutrophil activation and the potential for MPO-derived oxidant production; circulating mitochondrial DNA (mtDNA), which reflects mitochondrial injury and DAMP release and the ratio of reduced to oxidized glutathione (GSH/GSSG), which reflects thiol redox balance. These should be distinguished from direct molecular footprints of oxidation, including F2-isoprostanes, 4-hydroxynonenal (4-HNE), 3-nitrotyrosine and 8-hydroxy-2′-deoxyguanosine (8-OHdG) and markers of tissue response. The latter includes angiopoietin-2 (Ang-2) as an endothelial activation marker, soluble thrombomodulin (sTM) and syndecan-1 as markers of endothelial injury and glycocalyx shedding, soluble receptor for advanced glycation end products (sRAGE) and surfactant protein D (SP-D) as markers of epithelial injury, and interleukin-6 (IL-6) and neutrophil extracellular trap (NET)-related measurements as indicators of inflammatory and thromboinflammatory activity. Longitudinal sampling is particularly important in necrotizing pancreatitis, in which early sterile necrosis may transition to infected necrosis, and in sepsis, in which the dominant inflammatory and redox-active processes change over time. Integration of biology, sampling time and biological compartment may support patient stratification and assessment of target engagement in future redox-directed trials. Created in BioRender (access date 14 October 2026; Pollinger, K. (2026) https://BioRender.com/962w165).

A longitudinal, panel-based approach should currently be regarded as a conceptual and hypothesis-generating research framework, rather than a clinically validated strategy. An illustrative early plasma research panel could combine routinely available variables, such as platelet count and D-dimer, with investigational measures of endothelial injury (Ang-2, soluble thrombomodulin or syndecan-1), epithelial injury (sRAGE and/or SP-D), inflammatory activity (IL-6, IL-8 and soluble tumor-necrosis-factor (TNF) receptor-1), NET-related measurements and a carefully validated redox readout. Repeated sampling may be more informative than an isolated baseline value because the transition from sterile pancreatic inflammation to infected necrosis, or from early sepsis to immune dysfunction, changes the dominant biology over time. However, broader implementation is limited by assay availability, cost, turnaround time, pre-analytical variability, incomplete analytical standardization and the absence of validated clinical thresholds. Bronchoalveolar lavage fluid is closer to pulmonary biology and can distinguish alveolar biomarker profiles between ARDS subphenotypes, but its dilutional variability, regional sampling bias and limited feasibility in critically ill patients constrain routine use (Figure 5) [112]. Prospective studies must establish whether a defined panel can provide clinically actionable risk stratification, treatment selection or target-engagement assessment [110,115].

Retrospective latent-class analyses have repeatedly identified hyperinflammatory and hypoinflammatory ARDS subphenotypes. The hyperinflammatory pattern is characterized by greater shock, inflammatory biomarker concentrations, coagulopathy, fewer ventilator-free days and higher mortality [104,116]. It is clinically attractive to interpret this phenotype as redox-active, but this should not be assumed: standard plasma biomarker models do not directly measure compartment-specific ROS generation, antioxidant capacity or ferroptotic vulnerability. A redox-directed trial should therefore add pathway-proximal measurements rather than simply relabel an inflammatory subphenotype as oxidative stress. This distinction is critical for trial design. A hyperinflammatory phenotype may provide prognostic enrichment by identifying patients at greater risk of adverse outcome, but prognostic association does not establish predictive enrichment. Predictive enrichment requires prospective evidence that a biomarker-defined subgroup has a reproducible and treatment-specific response to a defined intervention. Therefore, neither hyperinflammatory classification nor increased inflammatory biomarker concentrations should be interpreted as evidence of a redox-defined, treatment-responsive phenotype.

The secondary Hydroxymethylglutaryl-CoA reductase inhibition with simvastatin in acute lung injury to reduce pulmonary dysfunction-2 (HARP-2) analysis provides an instructive example. Simvastatin was associated with improved survival in the hyperinflammatory subgroup but not in the hypoinflammatory subgroup, whereas the parent trial was neutral in the overall population [116]. This retrospective subgroup finding is hypothesis-generating rather than practice-changing. It does not establish statin therapy for hyperinflammatory ARDS and does not provide direct evidence that the hyperinflammatory phenotype predicts response to antioxidant or other redox-directed therapy. Prospective biomarker-enriched trials require a rapid and reproducible classifier, a prespecified treatment-by-phenotype interaction, and demonstrable target engagement in the relevant compartment [117].

NOX modulation, restoration of mitochondrial quality control, NRF2 activation, NET-directed approaches and ferroptosis-directed interventions remain experimental and are not currently applicable in routine ARDS care. Selective modulation of NOX4-associated injurious endothelial ROS may be beneficial in experimental systems, but indiscriminate the inhibition of NOX2 risks compromising phagocyte host defense. Restoring mitochondrial quality control—for example through PTEN-induced kinase 1 (PINK1)/Parkin-associated mitophagy—or enhancing NRF2-dependent stress responses may limit mtROS, inflammasome activation and pyroptosis in experimental sepsis-associated ARDS [118]. However, neither NOX4 modulation nor the strategies targeting mitophagy or NRF2 have established clinical efficacy, validated target engagement, or a defined therapeutic window in ARDS. The same interventions could be harmful if they are given after the dominant phase has shifted from injury control to repair, or if they blunt antimicrobial immunity in infected necrosis.

Ferroptosis provides a second example of a compelling but unproven target. System x_c−-dependent cystine uptake, glutathione synthesis and glutathione peroxidase 4 (GPX4) form a central defense against phospholipid hydroperoxides, while acyl-CoA synthetase long-chain family member 4 (ACSL4)-mediated incorporation of polyunsaturated fatty acids can increase susceptibility to lipid peroxidation [56,119]. Experimental studies support a contribution of ferroptosis to alveolar epithelial and endothelial injury; however, ferroptosis biomarkers are not validated for routine ARDS stratification, and neither iron chelation nor ferroptosis inhibition has established clinical efficacy in ARDS [119,120].

Evidence from vitamin C trials should be interpreted at the level of the intervention tested. In CITRIS-ALI, intravenous vitamin C did not improve the prespecified organ-dysfunction or biomarker endpoints in patients with sepsis and ARDS [121]. The larger LOVIT trial in vasopressor-dependent sepsis found an increased composite risk of death or persistent organ dysfunction with intravenous vitamin C [122]. These findings do not support unselected systemic vitamin C therapy in the studied populations. They do not, however, exclude redox-directed strategies that define a relevant pathway, biological compartment, treatment window and target-engagement marker before intervention.

The next translational step is a pathway-defined trial design: a biological target must be linked to a relevant sample type, an analytically robust biomarker threshold, a biologically plausible treatment window and host-defense safety monitoring. Clinical endpoints should be accompanied by pharmacodynamic evidence that the intended pathway changed in the relevant compartment. Multi-omic datasets and bedside physiological measures may help identify such patients, but their value must be established prospectively rather than inferred from association alone [117,123]. At present, clinical implementation is constrained by cost, assay and analytic standardization, data-processing reproducibility, turnaround time and the absence of external validation across independent cohorts. The evidence hierarchy, extent of human validation and current therapeutic status of the principal redox-sensitive pathways discussed in this review are summarized in Table 2.

## 6. Conclusions

ARDS is a syndrome of acute, diffuse inflammatory lung injury rather than a single biological disease [1]. Across sepsis and severe acute pancreatitis, oxidative stress is best viewed as a regulated but potentially maladaptive component of the host response [124]. ROS generated by NOX enzymes, mitochondria and activated leukocytes contribute to microbial defense and cellular signaling, yet sustained or mislocalized production can disrupt the endothelial and epithelial barriers, amplify inflammatory cell death and promote immunothrombosis [33,34].

Sepsis and severe acute pancreatitis provide complementary clinical contexts for redox-sensitive barrier failure. Their initiating signals and temporal evolution differ, but both may progress to mixed inflammatory states characterized by endothelial and epithelial injury, neutrophil activation, coagulation and microvascular dysfunction [20,28]. This context dependence argues against unselected antioxidant therapy and supports pathway-, compartment- and phase-specific translational studies. Whether reducing oxidant production is beneficial when lipid peroxidation, endothelial injury or mitochondrial dysfunction predominate remains a hypothesis requiring pathway-specific clinical validation. Such an intervention may be ineffective or harmful when antimicrobial oxidant functions, host defense or repair are required. The neutral or unfavorable results of unselected intravenous vitamin C therapy in critical illness underscore the need to define the biological target and treatment window rather than treating oxidative stress as a uniform systemic deficiency [33,122].

The next generation of studies should therefore link biomarker measurement to a predefined biological question. Repeated sampling of endothelial, epithelial, inflammatory, NET-associated and redox-related markers may identify patients with a coherent injury program. Such panels require analytical validation, clinically realistic sampling, external replication and prospective demonstration that they select a treatment-responsive subgroup [14,115]. Blood-based measures will remain practical, but their interpretation should be integrated with source of injury, disease phase, organ dysfunction and bedside physiology. Future interventional trials should be pathway- and phenotype-defined. They should specify whether the intended target is excessive NOX activity, mitochondrial injury, lipid peroxidation, glycocalyx loss, NET-driven immunothrombosis or another redox-sensitive process; they should also document target engagement and monitor effects on microbial clearance and repair. Adaptive platform designs could provide a setting in which a redox biomarker with clinically feasible turnaround is used prospectively to define a prespecified biological eligibility stratum for a pathway-matched intervention. Serial measurement would then be required to document target engagement, while ineffective or unsafe treatment arms could be discontinued without assuming that one intervention applies to all patients with ARDS [14,117]. This approach is consistent with current precision-medicine frameworks, which emphasize prospective validation of biologically coherent phenotypes, predictive enrichment and treatment-specific target engagement rather than retrospective subgroup discovery alone [125,126].

In conclusion, redox biology provides a unifying language for ARDS associated with sepsis and severe acute pancreatitis, but it does not justify a universal antioxidant prescription. Progress will depend on distinguishing physiological from damaging ROS signals, defining their source, compartment and timing, and pairing mechanistically credible interventions with biomarkers that can be used prospectively. This approach is more likely to yield clinically useful redox-directed therapy than further unselected trials.

## Figures and Tables

**Table 1:** Disease-specific redox context of sepsis- and severe acute pancreatitis-associated ARDS.

Domain	Sepsis-Associated ARDS	Severe Acute Pancreatitis-Associated ARDS	Potential Biomarker Readouts	Evidence Level	Key References
Initiating insult	Infection and pathogen-associated molecular patterns (PAMPs)	Acinar-cell injury, necrosis and damage-associated molecular patterns (DAMPs)	Sepsis: microbiological testing; pathogen-directed diagnostics. Pancreatitis: imaging-defined necrosis; microbiological assessment when infected necrosis is suspected.	Clinical definition and established disease biology	[28,59,76]
Predominant early inflammatory context	Pathogen-driven innate immune activation with variable microbial burden	Initially sterile systemic inflammation, metabolic stress and tissue necrosis	Sepsis: IL-6, IL-8, soluble TNF receptor-1, ferritin. Pancreatitis: CRP, IL-6 and inflammatory trajectories.	Human clinical phenotypes; molecular detail partly experimental	[28,41,59]
Major redox-active processes	Phagocyte NOX2 activity, myeloperoxidase-derived oxidants, iNOS-associated RNS and mitochondrial dysfunction	Acinar-cell calcium overload, mitochondrial injury, lipid peroxidation, impaired antioxidant defense and neutrophil-derived oxidants	Sepsis: MPO, GSH/GSSG, 3-nitrotyrosine, mtDNA. Pancreatitis: GSH/GSSG, F2-isoprostanes, 4-HNE/MDA, mtDNA.	Predominantly experimental; supported by associative human data	[28,33,39,41,65,68,82,83,84,85,86]
Mitochondrial dysfunction and metabolic failure	Inflammatory nitric-oxide signaling, impaired electron transport, mtROS generation and release of mitochondrial DAMPs	Acinar-cell calcium overload, mitochondrial permeability transition, ATP depletion and necrotic cell death	Circulating mtDNA may reflect a DAMP-rich state but is not a lung-specific or treatment-selective marker.	Experimental causality; human evidence mainly associative	[65,68,82,98]
Link to alveolar–capillary barrier failure	Systemic endothelial activation, microvascular dysfunction, leukocyte recruitment and immunothrombosis	Circulating DAMPs, cytokines, oxidized lipids, pancreatic injury signals and pulmonary microvascular activation	Ang-2, soluble thrombomodulin and syndecan-1 as endothelial injury markers; sRAGE and SP-D as epithelial injury markers.	Human association with experimental mechanistic support	[17,19,24,28,33,41,89]
Neutrophils, NETs and immunothrombosis	Neutrophil activation and NET-related thromboinflammation may amplify barrier injury; NET markers are associated with severity	Experimental NET formation aggravates acute pancreatitis and remote-organ injury; human NET markers are associated with pancreatitis severity	Cell-free DNA, MPO–DNA complexes and citrullinated histone H3 are indirect NET-related readouts; none is validated for treatment selection.	Human observational evidence plus animal intervention studies	[40,70,71,92]
Characteristic temporal transition	Infection control, persistent inflammation and later immune dysfunction may alter the dominant redox program	Early sterile necrosis may progress to intestinal-barrier failure, infected necrosis and superimposed PAMP-driven sepsis; after this transition, the inflammatory and redox phenotypes increasingly overlap with sepsis-associated ARDS	Serial measurements of inflammatory, tissue-injury and redox-related markers; microbiological reassessment where clinically indicated.	Clinical natural history; mechanistic transitions partly experimental	[28,59,76]
Available human biomarker evidence	Inflammatory, endothelial, NET-related and mitochondrial markers may support risk stratification or biological enrichment	Inflammatory and oxidation-related markers correlate with pancreatitis severity and pulmonary complications	Associations do not establish the dominant causal pathway in an individual patient and do not select patients for redox-directed therapy.	Observational clinical evidence; no validated predictive biomarker	[40,83,84,92,99]
Clinical priorities and implication for redox-directed therapy	Rapid identification and control of infection, appropriate antimicrobials and preservation of antimicrobial oxidant functions; redox-directed therapy remains unproven	Assessment of persistent organ failure and surveillance for infected necrosis; enteral nutrition and, when infection is confirmed, delayed minimally invasive source control; broad antioxidant therapy remains unsupported	No biomarker is validated for selecting patients for redox-directed therapy.	Guideline-supported clinical management; no validated redox-directed treatment or biomarker-guided strategy	[28,59,76]

Sepsis- and severe acute pancreatitis-associated ARDS converge on redox-sensitive alveolar–capillary barrier failure but differ in their initiating signals, temporal evolution and therapeutic constraints. Clinical descriptions refer to severe acute pancreatitis and, where reported, clinically defined ARDS; mechanistic statements derive predominantly from experimental models of pancreatitis-associated lung injury. Abbreviations: PAMPs, pathogen-associated molecular patterns; DAMPs, damage-associated molecular patterns; NOX2, NADPH oxidase 2; NADPH, nicotinamide adenine dinucleotide phosphate; IL, interleukin; TNF, tumor necrosis factor; CRP, C-reactive protein; MPO, myeloperoxidase; GSH/GSSG, reduced/oxidized glutathione; mtDNA, mitochondrial DNA; 4-HNE, 4-hydroxynonenal; MDA, malondialdehyde; Ang-2, angiopoietin-2; sRAGE, soluble receptor for advanced glycation end products; SP-D, surfactant protein D; NETs, neutrophil extracellular traps; iNOS, inducible nitric-oxide synthase; RNS, reactive nitrogen species; ARDS, acute respiratory distress syndrome.

**Table 2:** Evidence hierarchy for redox-sensitive pathways implicated in sepsis- and severe acute pancreatitis-associated ARDS.

Pathway	Proposed Effect on the Alveolar–Capillary Barrier	Disease Context	Type of Evidence	Human Validation	Therapeutic Status	Current Clinical Status
Lung-protective ventilation and prone positioning	Limits ventilator-associated injury and improves gas exchange.	Established ARDS	Randomized trials and guidelines support use in appropriately severe ARDS [3].	Established.	Procedure- and patient-specific risks.	Established supportive care
Source control, enteral nutrition and delayed step-up treatment	Controls infected necrosis and limits persistent inflammatory drive.	Severe acute pancreatitis	Clinical studies and guidelines support infection surveillance, enteral nutrition and delayed minimally invasive treatment when indicated [28,97].	Established for clinical management.	Timing and procedural risks.	Established when clinically indicated
Intravenous vitamin C	Broad systemic antioxidant supplementation.	Sepsis with ARDS/vasopressor-dependent sepsis	CITRIS-ALI did not improve prespecified primary outcomes [121]; LOVIT showed an unfavorable composite outcome [122].	Evaluated in randomized trials without consistent benefit.	Potential harm in unselected sepsis.	Not recommended as routine redox therapy
Statin therapy in hyperinflammatory ARDS	Pleiotropic endothelial and anti-inflammatory effects.	ARDS subphenotypes	Overall HARP-2 trial neutral; secondary subgroup signal hypothesis-generating [116].	No validated prospective enrichment strategy.	No rapid validated classifier; no confirmatory trial.	Not established
NOX2/NOX4-derived oxidants	NOX2 supports antimicrobial phagocyte activity; sustained NOX4-associated signaling may promote endothelial junctional disruption and permeability.	Sepsis-associated ARDS; experimental acute lung injury	Cellular and animal studies; observational human studies of neutrophil activation and endothelial injury [19,21,38,39].	No causal role for an individual NOX isoform established in human ARDS.	NOX modulation remains preclinical; NOX2 inhibition may compromise host defense.	NOX2 inhibition may compromise host defense.
Mitochondrial dysfunction and mtROS	Oxidant generation, bioenergetic failure, mitochondrial DAMP release and endothelial/epithelial stress.	Sepsis- and pancreatitis-associated ARDS	Cellular and animal studies; human observational studies of mitochondrial dysfunction and circulating mtDNA [65,66,67,68,98].	Association with illness severity and outcome; no validated causal or treatment-selection role.	Mitochondria-directed strategies and mitophagy enhancement remain preclinical.	Mistimed modulation may impair adaptive stress responses or repair.
NRF2-dependent antioxidant response	May strengthen antioxidant defenses and limit oxidant-associated tissue injury.	Primarily experimental pancreatitis and sepsis-associated lung injury	Predominantly cellular and animal studies [32,118].	No established human target-engagement marker or therapeutic validation in ARDS.	NRF2 activation remains investigational.	Potential interference with host defense and phase-specific repair.
NLRP3 inflammasome activity and pyroptosis	May amplify cytokine release, inflammatory cell death and barrier injury.	Sepsis- and pancreatitis-associated lung injury	Cellular and animal studies; human inflammatory-marker associations [57,85,86].	No causal validation in individual patients with ARDS.	Inflammasome-directed therapy remains experimental.	Broad inhibition may compromise microbial clearance.
NET formation and immunothrombosis	Histones, proteases and oxidants may injure endothelial and epithelial cells; NET scaffolds promote coagulation.	Sepsis- and pancreatitis-associated ARDS	Human observational studies plus animal intervention studies [70,71,92].	NET-related markers associate with disease severity; no validated treatment-selection biomarker.	NET-directed interventions are not established; indiscriminate suppression may impair host defense.	Indiscriminate suppression may impair host defense.
Lipid peroxidation and ferroptosis	Membrane lipid damage and regulated cell death may impair endothelial and epithelial barrier integrity.	Experimental ARDS; pancreatitis-associated lung injury	Predominantly cellular and animal studies [56,58,83,119].	Ferroptosis markers are not validated for routine ARDS stratification.	Iron chelation and ferroptosis inhibition have no established clinical efficacy in ARDS.	No established efficacy; risks of nonselective intervention unknown.
Pancreas–gut–lung crosstalk	DAMP release, intestinal-barrier dysfunction, bacterial translocation and circulating inflammatory signals may amplify pulmonary injury.	Necrotizing pancreatitis-associated ARDS	Experimental models, translational studies and clinical association data [87,88,89,90,91,92,94,95,96].	Clinical evidence supports the relevance of infected necrosis and intestinal-barrier failure; individual causal lung pathways remain unproven.	Supportive care, infection surveillance and source control are guideline-based; redox-directed approaches remain unproven.	Clinical timing and infection status determine appropriateness of intervention.

Abbreviations: ARDS, acute respiratory distress syndrome; CITRIS-ALI, Vitamin C Infusion for Treatment in Sepsis-Induced Acute Lung Injury; LOVIT, Lessening Organ Dysfunction with Vitamin C; HARP-2, Hydroxymethylglutaryl-CoA reductase inhibition with simvastatin in acute lung injury to reduce pulmonary dysfunction-2; NOX2/NOX4, NADPH oxidase 2/4; mtROS, mitochondrial reactive oxygen species; DAMP, damage-associated molecular pattern; NRF2, nuclear factor erythroid 2-related factor 2; NLRP3, NOD-like receptor family pyrin domain-containing 3; NETs, neutrophil extracellular traps.

## Data Availability

No new data were created or analyzed in this study. Data sharing is not applicable to this article.
